# Ultrasensitive Detection of Ochratoxin A With a Zeolite Imidazolate Frameworks Composite–Based Electrochemical Aptasensor

**DOI:** 10.3389/fchem.2022.858107

**Published:** 2022-04-06

**Authors:** Xiao Ni, Yuyan Zhang, Chuhan Xue, Xiaojun Chen

**Affiliations:** ^1^ College of Chemistry and Molecular Engineering, Nanjing Tech University, Nanjing, China; ^2^ Shanghai Pudong New District Jincai High School, Shanghai, China; ^3^ Jiangsu Key Laboratory of Molecular Biology for Skin Diseases and STIs, Nanjing, China

**Keywords:** zeolite imidazolate frameworks (ZIF-8), electrochemical aptasensors, mycotoxin, ochratoxin A (OTA), signal amplification strategy

## Abstract

Ochratoxin A (OTA) is a harmful mycotoxin, which is mainly secreted by *Penicillium* and *Aspergillus* species. In this work, an electrochemical aptasensor is presented for OTA detection based on Au nanoparticles (AuNPs) modified zeolite imidazolate frameworks (ZIFs) ZIF-8 platform and duplex-specific nuclease (DSN) triggered hybridization chain reaction (HCR) signal amplification. G-quadruplex-hemin assembled HCR nanowire acted as a nicotinamide adenine dinucleotide (NADH) oxidase and an HRP-mimicking DNAzyme. Besides, thionine (Thi) was enriched as a redox probe for signal amplification in this pseudobienzyme electrocatalytic system. Under the optimal conditions, the analytical response ranged from 1 to 10^7^ fg ml^−1^ with a detection limit of 0.247 fg ml^−1^. Furthermore, the aptasensor was proven to be applied in real wheat samples with a recovery between 96.8 and 104.2%, illustrating the potential prospects in practical detection.

## Introduction

Ochratoxin is one of the secondary metabolism substances of ochra and penicillium fungi, which includes seven types of homologous structure compounds. Under the classification of WHO’s International Agency for Research on Cancer, ochratoxin A (OTA) is known as the highest-toxic-ranking category, marking as Level 2B carcinogen (Lee and Ryu, 2017). It can threaten the health of most mammalian species in liver, kidneys, and immune system. OTA contamination occurs in most crop types, such as corn and wheat, and causes accumulation in livestock (Lee and Ryu, 2015). Besides, due to the high chemical stability, OTA rarely has quantity loss in the transportation and storage or lower risk of toxic hazards through additional manufacturing procedures (Abrunhosa et al., 2002). Hence, OTA can even be found in people’s body since they eat the contaminated food (García-Campaña et al., 2012). Fully taking into account the potential toxicity, firm standards for residue harmful substance have been established by European regulators and the maximum content of OTA in both farm and sideline products shall not exceed 10 µg kg^−1^. In general, the concentration of OTA often demonstrates low levels especially in the early stage. With the increase in health consciousness, associated research for detection of OTA in foodstuff has garnered particular attention.

Up to now, the universal analysis for OTA detection is largely concentrated in advanced instrumental methods, including high-performance liquid chromatography (HPLC), HPLC-fluorescence detector (HPLC-FLD), ELISA, and immunochromatographic assay (Pittet and Royer, 2002; Cho et al., 2005; Giovannoli et al., 2014; Zhang et al., 2017; Sun Zhichang et al., 2018; Armstrong-Price et al., 2020). Although these chromatography and immunoassays have high precision and are selective during external interference, there still exist inescapable disadvantages such as high-cost expenses, complicated operation requirements and strict experimental conditions, and difficulty to conduct field testing. Therefore, the upcoming challenge is to have higher sensitivity and cost reduction advantages in OTA detection to realize application in practice. Compared with the aforementioned methods, electrochemical methods are preferable due to the low cost and short response, and are suitable for miniaturization in practical applications. Thereafter, various electrochemical biosensors for OTA have arisen, mainly involving immunosensors, capacitance biosensors, photo-electrochromic biosensors, aptasensors, etc. (Chen et al., 2012; Bougrini et al., 2016; Duan et al., 2017; Ying et al., 2018). Among them, the electrochemical aptasensors have been attracting more and more attention since they combine the advantages of electrochemical methods and aptamers.

Alternatively, aptamers have a favorable ascendancy in stability, affinity, and output, which make them obtain very high commercial availability (Cheow and Han, 2011; Han et al., 2012). Ever since the first time it was reported that the aptamer was applied for OTA detection by Cruz-Aguado’s team (Cruz-Aguado and Penner, 2008), diverse OTA electrochemical aptasensors have been widely introduced. Hairpin-DNA aptamer toward OTA was first reported by Zhang et al. with an analytical response varying from 1.0 to 20 pg ml^−1^ (Zhang et al., 2012). Moreover, one recent work proposed a new model of OTA aptasensor, based on self-assembly between SH-aptamer and gold layer deposition, allowing to show wide-dynamic-range capabilities (1 × 10^−5^–10 nM). Researchers also applied the aptasensor to investigate OTA in red wine samples (Yang et al., 2019). Although the sensitivity of these sensors has fulfilled OTA detection with a low content, development of novel aptasensors from various angles is still imperative.

To further make the best use of electrochemical aptasensors, electrode materials are crucial to enhance the sensitivity and stability. A series of nanomaterials were introduced to bring larger active surface and higher electrical conductivity for amplifying the response to weak signals (Manna and Raj, 2018). Metal-organic frameworks (MOFs), characteristic of high crystalline structure, have shown extensive application prospect in catalysis, medical science, biological toxicity testing, etc. (Wang P.-L. et al., 2019). However, bare MOFs did worse in constructing electrochemical aptasensors as they often conduct electricity poorly. Therefore, various MOF composites with metal nanoparticles, carbon nanostructures, and conductive polymers have been assembled for a wide application in electrochemical field (Zhang et al., 2020). In addition, zeolitic imidazolate frameworks (ZIFs) belonging to one of those prove to have special structure of zeolite, which is characteristic of both thermal and chemical stability (Keskin, 2011). Because of the aforementioned advantages, ZIFs composite was used as a carrier in this work to load biomaterials. At present, the quest for ZIF-based aptasensors is still under way (Hao et al., 2019; Salandari-Jolge et al., 2021), and significantly, few of them have been applied in aspect of OTA detection.

**SCHEME 1:**
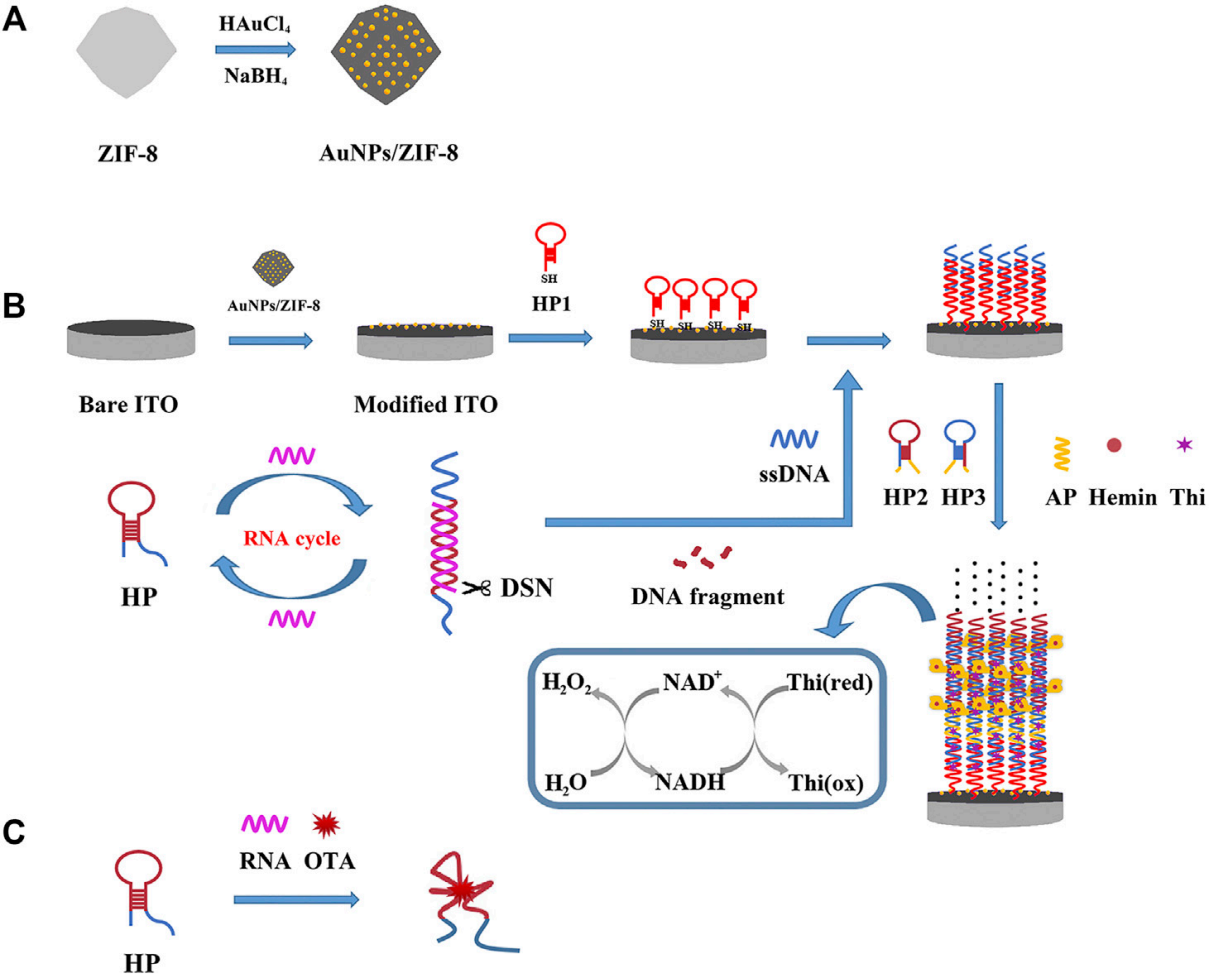
Schematic illustration of the principle of the electrochemical aptasensor: **(A)** fabrication of the AuNPs/ZIF-8, **(B)** DSN-assisted signal amplification strategy in the absence of OTA, and **(C)** selective recognition of HP with OTA.

With this idea in mind, a heterogeneous composite of Au nanoparticles (AuNPs)/ZIF-8 was selected and served as a conductive platform for supporting and sensing, by implanting ultrafine AuNPs into the highly regular ZIF-8 while the dodecahedral structure was unchanged. At the same time, in attempt to boost the number of recycling oligonucleotides for signal amplification, duplex-specific nuclease (DSN) was adopted. It can identify and digest DNA strand from a DNA/RNA hybrid to free RNA and achieving recovery (Qiu et al., 2015). The schematic illustration of this work is displayed in Scheme 1. In the absence of OTA, hairpin aptamer (HP) hybridized with RNA strand, which had an unmatched ssDNA on 3′-end. DNA strand in the duplex can be shorn by DSN to release RNA and trigger more cycles of DSN reaction. A short strand named ssDNA could be released to open HP1 assembled on the electrode, which initiated a HCR on the AuNPs/ZIF-8 modified electrode surface. The remaining parts in HP2 and HP3 combined with Hemin molecules to form G-quadruplex-hemin DNAzymes. In the catalytic reaction, thionine (Thi) was adopted as a redox probe and nicotinamide adenine dinucleotide (NADH) worked as coenzyme to further amplify the electrochemical signal (Yuan et al., 2014). Otherwise, with certain amount of OTA, HP tended to interact with OTA while RNA strands are released. HP/OTA failed to release ssDNA and trigger the subsequent HCR reactions, and then a weaker redox signal was recorded in comparison with circumstances when no OTA exists. In this perspective, the relationship between OTA content (C_OTA_) and current signal has a negative linear correlation instead of a linear one.

## Experimental

### Materials and Instrumentation

Zinc (II) nitrate hexahydrate [Zn(NO_3_)_2_·6H_2_O], 2-methyl imidazole (2-MI), and sodium borohydride (NaBH_4_) were purchased from Aladdin. Chloroauric acid hydrated (HAuCl_4_·4H_2_O) was obtained from Beijing HWRK. NADH, Thi, and hemin were obtained from Aldrich. Bovine serum albumin (BSA) and Tris (2-carboxyethyl) phosphine hydrochloride (TCEP-HCl) were purchased from Baoman Biotech. Co., Ltd. Phosphoric acid buffer solution (PBS, 0.1 M, pH 7.0) was prepared by regulating the amount of Na_2_HPO_4_ and NaH_2_PO_4_ and used as electrolyte. PBS containing 5.0 mM [Fe(CN)_6_]^3−/4−^ was used for cyclic voltammetry (CV) and electrochemical impedance spectroscopy (EIS) analysis. Hemin solution was prepared by 0.15 mM hemin, 0.25 mM HEPES, 0.20 mM KCl, 2 mM NaCl, and 1 mM DMSO. OTA was obtained from PriboLab. DSN and the buffer were obtained from Evrogen. All functional group-modified oligonucleotides were purified by Sangon Biotechnology Co., Ltd. Indium tin oxide (ITO)–coated electrodes (coating thickness 180 ± 25 nm, sheet resistance <15 U/cm^2^) were purchased from Kaivo Electronic Components Co., Ltd. All reagents were prepared with ultrapure water. All other chemicals were analytical grade and used without further purification. The oligonucleotide sequences are listed in Table 1.

**TABLE 1:** Oligonucleotides used in this work

Oligonucleotide	Sequence (from 5′ to 3′)
HP	HS-C6- TTT TTT GAT CGG GTG TGG GTG GCG TAA AGG GAG CAT CGG ATC AAT CCG TCG AGC AGA GTT CCA TGT GTA GAT AGC TTA
HP1	HS-C6- CCA TGT GTA GAT CAG ACT ATT CGA TTA AGC TAT CTA CAC ATG G
HP2	AGG GCG GGT GGG TGT TTA AGT TGG AGA ATT GTA CTT AAA CAC CTT CTT CTT GGG T
HP3	TGG GTC AAT TCT CCA ACT TAA ACT AGA AGA AGG TGT TTA AGT TGG GTA GGG CGG G
AP	AAC TCT GCT CGA CGG ATT AGA AGA AGG TGT TTA AGT
RNA	U CCG AUG CUC CCU UUA CGC CAC CCA CAC CCG AUC

Note: The red font in HP is the OTA aptamer. Letters of the same color (blue, green, and brown) are respectively complementary to each other. The stem part in HP, HP1, HP2, and HP3 was marked with underlines.

Electrochemical measurements including differential pulse voltammetry (DPV), CV, and EIS were conducted on a CHI 660E electrochemical workstation (Chenhua Instrument). The morphology of the nanostructures was investigated by scanning electron microscopy (SEM; Zeiss Sigma 300) and transmission electron microscope (TEM; JEOL JEM-F200). Elemental mapping and energy dispersive spectrometry (EDS) were characterized on TEM (Hitachi S4800). The X-ray diffraction (XRD) pattern was recorded in the 2θ scan range from 5 to 40° using Cu Kα radiation with wavelength (λ) of 0.154 nm. Fourier-transform infrared resonance (FTIR) spectra were conducted on a Nicolet iS5 (Thermo Fisher Scientific) instrument using KBr pellet method. The chemical states were measured by X-ray photoelectron spectroscopy (XPS; Thermo Kalpha). The nitrogen adsorption–desorption isotherms were recorded by a gas sorption analyzer (ASAP2460), and the specific surface area was estimated by Barrett–Emmett–Teller (BET) theory. Agarose gel electrophoresis (AGE) analysis was conducted in 1× TAE buffer under 120 V for 40 min, in which the concentration of agarose was 2%. The gel stained by ethidium bromide (EB) was then separated to have images under gel imaging system (Bio-Rad). The 50–500 bp DNA Ladder (Solarbio) was used as marker to analyze DNA bands.

### Synthesis of ZIF-8

ZIF-8 was prepared according to a previous literature with a slight adjustment (Torad et al., 2013). Then 1 mmol Zn(NO_3_)_2_·6H_2_O and 8 mmol 2-MI were respectively added to 10 ml methanol to form two clear solutions. They were then mixed into one beaker and let stand overnight for stratification. The white precipitated ZIF-8 was prepared in steps of methanol washing and vacuum drying.

### Synthesis of AuNPs/ZIF-8

AuNPs/ZIF-8 was prepared according to a previous report (Wang Y. et al., 2019). ZIF-8 (0.2 g) was taken and 20 ml of 10 mM HAuCl_4_ solution was added into a 50-ml beaker. To guarantee sufficient reaction, the solution was first ultrasonicated for 10 min and stirred vigorously for another 12 h. Then, 0.2 M NaBH_4_ solution was quickly added and stirred for another 30 min. The resulting AuNPs/ZIF-8 was prepared in steps of water washing and vacuum drying.

### Fabrication of AuNPs/ZIF-8 Modified Electrode

First, ITO electrode underwent ultrasonic cleaning in acetone and then successively treated with ethanol and distilled water, and then dried with nitrogen for further use. A region of space for electrochemical testing was confined to a 3-mm-diameter circular controlled by tape punching. Then, 10 µl of 1 mg ml^−1^ AuNPs/ZIF-8 dispersion was obtained and dribbled on the electrode surface. It was left to stand under room ambience to dry naturally and then stored at 4°C, which was designated as AuNPs/ZIF-8/ITO.

### Process of Aptasensing

Before aptasensing, 20 µl of HP, HP1, HP2, or HP3 solution with a concentration of 4 µM was brought to a 95°C thermostatic water bath for 5 min and then cooled down to room temperature prior to use. Significantly, since HP and HP1 are –SH group modified, to reduce the formation of disulfide bond, per 100 µl of solution was added with 0.1 µl of 100 mM TCEP-HCl after activation. All DNA sequences were diluted with PBS and RNA sequence was diluted with TE buffer. Twenty microliters of different concentrations of HP1 was immersed on the AuNPs/ZIF-8/ITO surface at room temperature for 2 h. In this procedure, thiolated HP1 bound to the electrode surface via Au–S bonding. After that, the electrode was rinsed with distilled water and then incubated with 0.25 wt.% BSA solution for 30 min to block the residue active sites on electrode surface.

After that, the electrode was immersed in 20 µl of mixture (containing 7 µl of 2 µM HP, 1 µl of OTA with different concentrations, 1 µl of 10× DSN buffer, 1 µl of 2 µM RNA, and 0.1 U DSN) and reacted at 50°C for 30 min. This process was aimed at activating DSN and cleaving DNA–RNA duplex. Then, 10 µl of 2× DSN STOP solution was added for another 5 min. At this time, the released DNA had been digested and ssDNA had been generated. A part of HP1 strands on electrode surface could hybrid with ssDNA after incubating with the aforementioned mixture at room temperature. After thoroughly rinsed with PBS, the ssDNA/HP1/AuNPs/ZIF-8/ITO electrode was incubated with 10 µl mixture containing 1 µM HP2, HP3, and AP, 0.25 mM Thi, and 0.15 mM hemin to initiate HCR reaction (Sun Xiaofan et al., 2018). After the incubation, the hemin/G-quadruplex came to generate and acted as a horseradish peroxidase (HRP) mimicking enzyme for NADH oxidation to NAD^+^ with the aid of dissolved O_2_. During this process, Thi worked as an electron mediator and a dramatically amplified current signal could be observed. DPV measurements were carried out in 10 ml PBS and the addition of NADH was 3 mM. The potential range came between −0.4 and 0 V.

## Results and Discussion

### Characterization of AuNPs/ZIF-8

**FIGURE 1:**
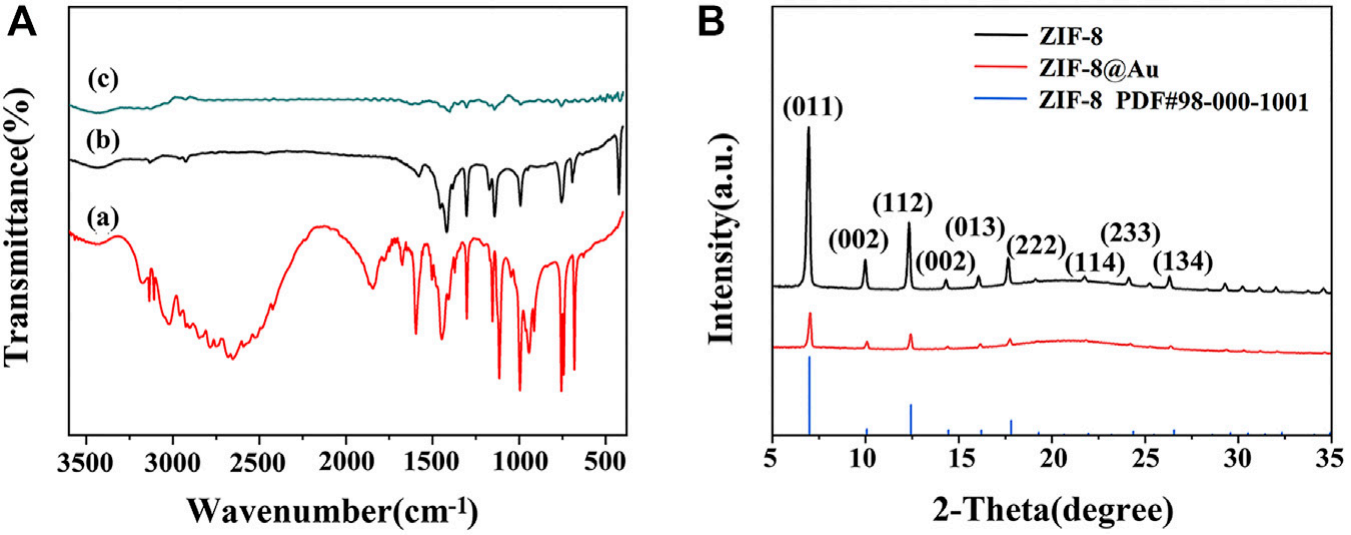
**(A)** FTIR spectra of (a) 2-MI, (b) ZIF-8, and (c) AuNPs/ZIF-8. **(B)** XRD patterns of ZIF-8 and AuNPs/ZIF-8.

Surface functional groups and formation of zinc nuclear imidazole coordination polymer were observed by FTIR spectra. As depicted in Figure 1A, both precursor ZIF-8 (curve b) and AuNPs/ZIF-8 (curve c) showed a C=N stretching vibration at 600 cm^−1^ and an aliphatic C–H stretch at 3,129 and 2,929 cm^−1^, which is caused by methyl and imidazole ring of imidazole ligands (Yang et al., 2015; Zhang et al., 2011). Besides, the band at 400 cm^−1^ is characterized by Zn–N stretch, demonstrating that Zn^2+^ was coordinated with N atom in the imidazole ring, and the structure of the ligand was intact (Zhang et al., 2016). Compared with FTIR spectra of imidazole ligands (curve a), observations cannot be made at 1,845 or 2,648 cm^−1^, which should be credited with N–H vibration and the N–H–N hydrogen bond absorption. The disappearance of the two strong bands shows the deprotonation of 2-methyl imidazole in the preparation process. It is known that there are no characteristic peaks for Au in the IR region and the analyzed FTIR result of AuNPs/ZIF-8 coincides well with that conclusion (Wang, Y., et al., 2019).

XRD patterns also authenticated the preparation of ZIF-8 and AuNPs/ZIF-8, as illustrated in Figure 1B. The presence of diffraction peaks with high intensity demonstrated that the synthesized products all had a crystalline structure. The characteristic peaks at 7.4°, 10.4°, 12.3°, 16.5°, 18.1°, and 26.8° corresponded to the crystal plane of (011), (002), (112), (013), (222), and (134) belonging to ZIF-8, which is consistent with previous reports (Park et al., 2006). In addition, the sharp peaks appearing at 7.4° and 12.3° indicated that the synthesized ZIF-8 structure was highly crystalline. Shown as the red curve in Figure 1B, after encapsulation of AuNPs, the main diffraction peaks of ZIF-8 remained unchanged, which can be attributed to the small sizes and high dispersion of AuNPs (Zhang et al., 2018).

**FIGURE 2:**
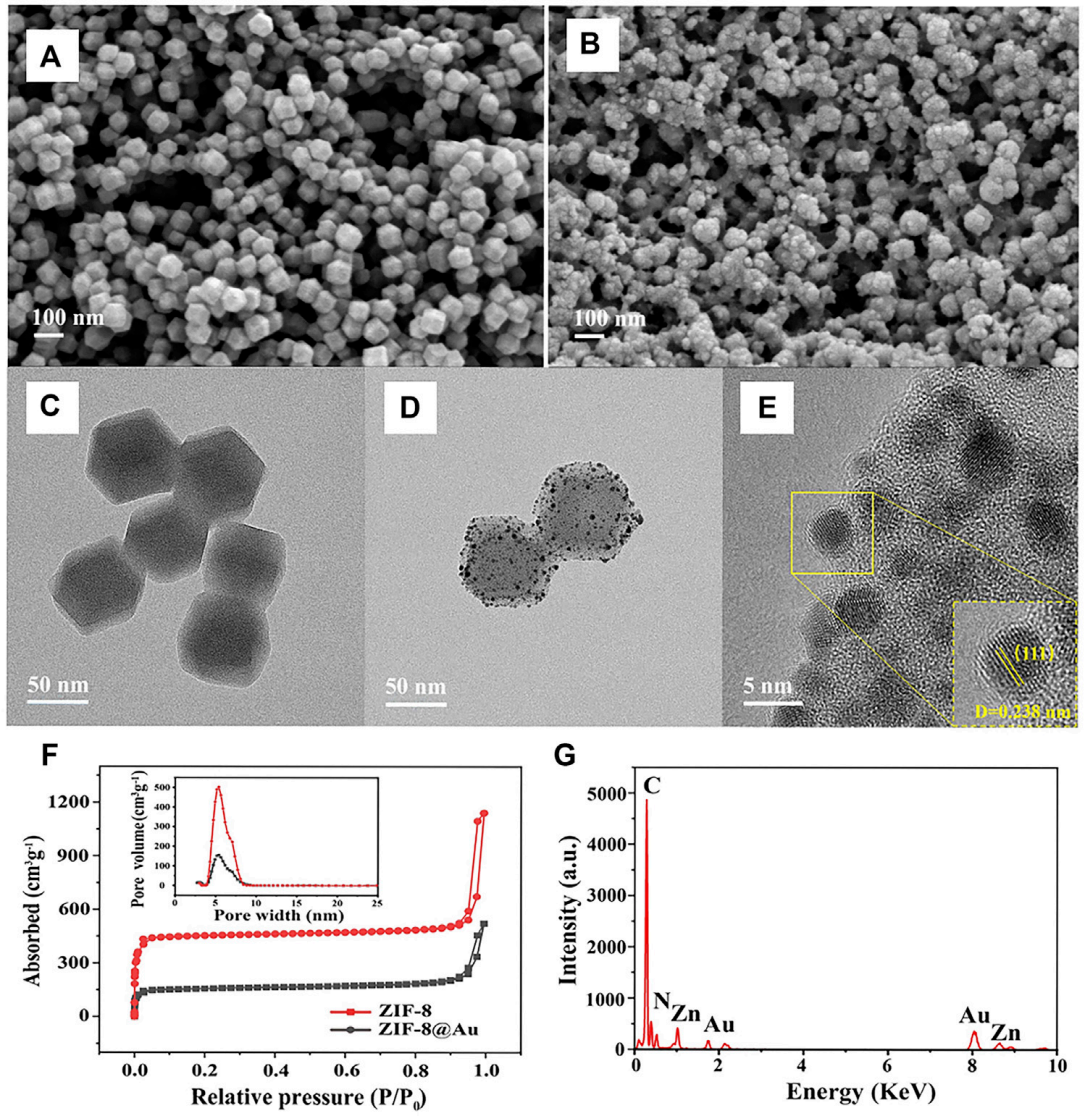
SEM images of **(A)** ZIF-8 and **(B)** AuNPs/ZIF-8. TEM images of the **(C)** ZIF-8 and **(D)** AuNPs/ZIF-8. **(E)** HRTEM image of AuNPs/ZIF-8. **(F)** N_2_ adsorption–desorption isotherms of ZIF-8 and AuNPs/ZIF-8 (inset: pore size distribution). **(G)** EDS spectrum of AuNPs/ZIF-8.

The structure and morphology of ZIF-8 and AuNPs/ZIF-8 were characterized by TEM and SEM, respectively. As seen in Figures 2A,B, typical uniform rhombic dodecahedron shape of ZIF-8 was preserved entirely after AuNP modification, and the particle size was found to increase from 65 nm (Figure 2C) to 80 nm (Figure 2D). Since AuNPs were produced *in situ* on the surface of ZIF-8 particles, some AuNPs uniformly scattered over the surface of ZIF-8 particles and others were implanted inside of ZIF-8, as seen in Figures 2D,E. To evaluate the pore structure and surface area before and after AuNPs interspersing into ZIF-8, N_2_ adsorption–desorption isotherms were performed (Figure 2F). According to the IUPAC classification, two hysteresis loops of Type-I were observed, indicating the presence of microporous structure of ZIF-8 and AuNPs/ZIF-8 materials (Sun et al., 2019). The surface area of AuNPs/ZIF-8 was calculated to be 628.8 ± 2 m^2^ g^−1^, which was much smaller than that of ZIF-8 (1,880.1 ± 2 m^2^ g^−1^). The pore size of AuNPs/ZIF-8 (5.38 nm) was a little smaller than that of the parent ZIF-8 (5.42 nm), and the total pore volume also decreased by about 68%. The result illustrated that AuNPs have implanted into the pores of ZIF-8 (Li Ying et al., 2021). Meanwhile, TEM images of Figure 2D demonstrated the uniform distribution of AuNPs into ZIF-8 surface, which was further confirmed in Figure 2E. The EDS result of Figure 2G verified the existence of Zn, C, N, and Au elements in an AuNPs/ZIF-8 singular particle.

**FIGURE 3:**
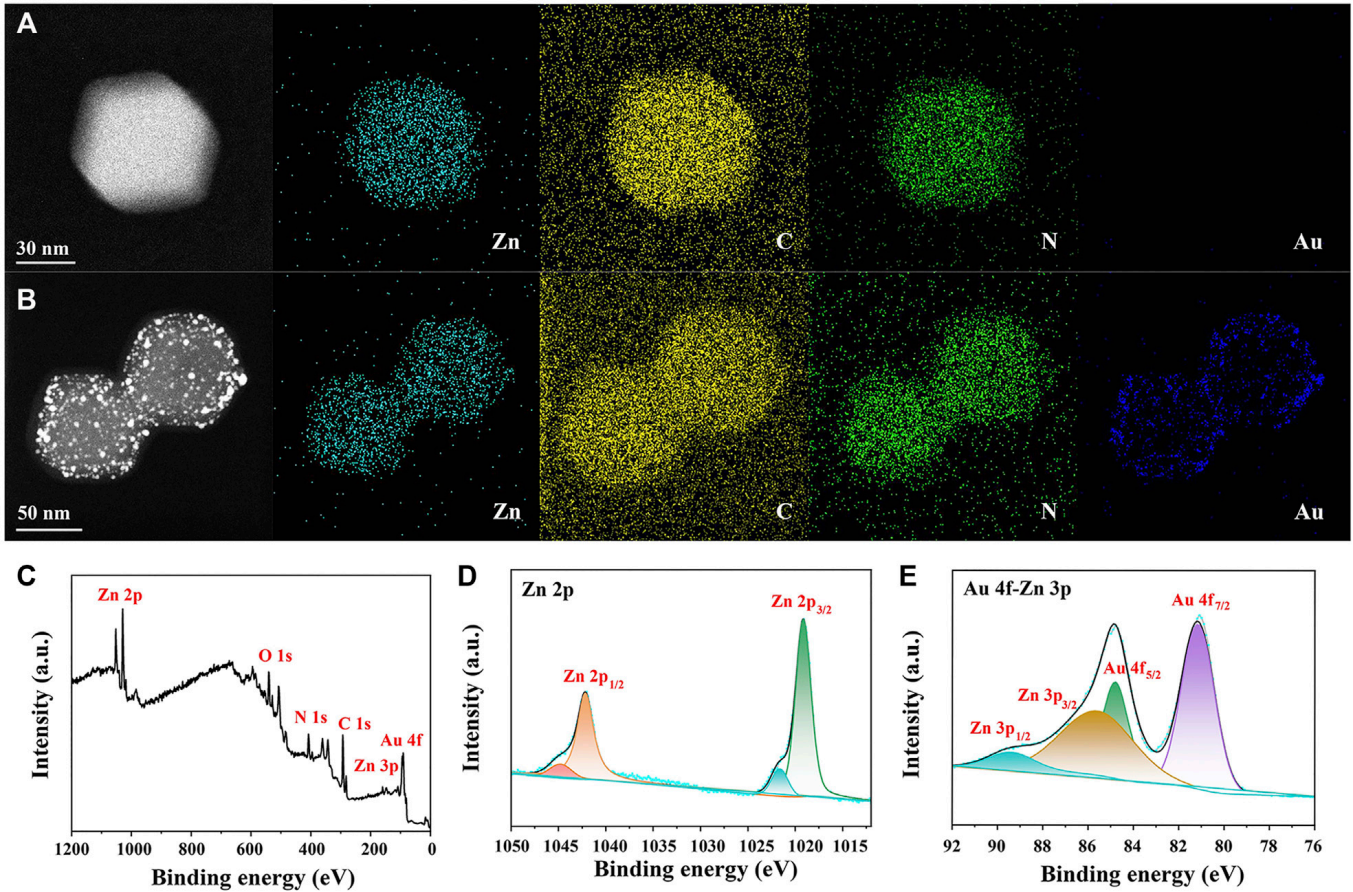
EDS mapping of **(A)** ZIF-8 and **(B)** AuNPs/ZIF-8. **(C)** XPS survey spectrum, high-resolution XPS spectra of **(D)** Zn 2p and **(E)** Au 4f-Zn 3p of AuNPs/ZIF-8.

Elementary mapping was also applied for confirming AuNP distribution in AuNPs/ZIF-8. Compared with Figure 3A of ZIF-8, uniformly distributed AuNPs could be observed on ZIF-8 surface (blue dots in Figure 3B). As shown in Figure 3C, XPS measurements were conducted to determine the valance states of different atoms in AuNPs/ZIF-8. In Figure 3D, the high-resolution Zn 2p spectrum in AuNPs/ZIF-8 split into doublet peaks due to the spin–orbit coupling effect, corresponding to Zn 2p_1/2_ and Zn 2p_3/2._ Meanwhile, Zn 2p spectrum was deconvoluted into four peaks, which were assigned to Zn–N (1,019.22 eV, 1,042.16 eV) and Zn–O (1,021.69 eV, 1,044.63 eV), respectively, which indicated the presence of +2 oxidation state of Zn (Tuncel and Ökte, 2021). As shown in Figure 3E, the XPS spectrum of Au 4f overlapped with that of Zn 3p, which could be deconvoluted into four peaks of Au 4f_7/2_ at 81.18 eV, Au 4f_5/2_ at 84.80 eV, Zn 3p_3/2_ at 85.64 eV, and Zn 3p_1/2_ at 89.63 eV, respectively (Gao et al., 2020). It can be noted that the intensity of Au was lower than that of Zn^2+^, which can be attributed to the sub-participation of AuNPs, and the obtained results were consistent with EDS analysis (Figure 2G).

### Feasibility of the Amplification Strategy

**FIGURE 4:**
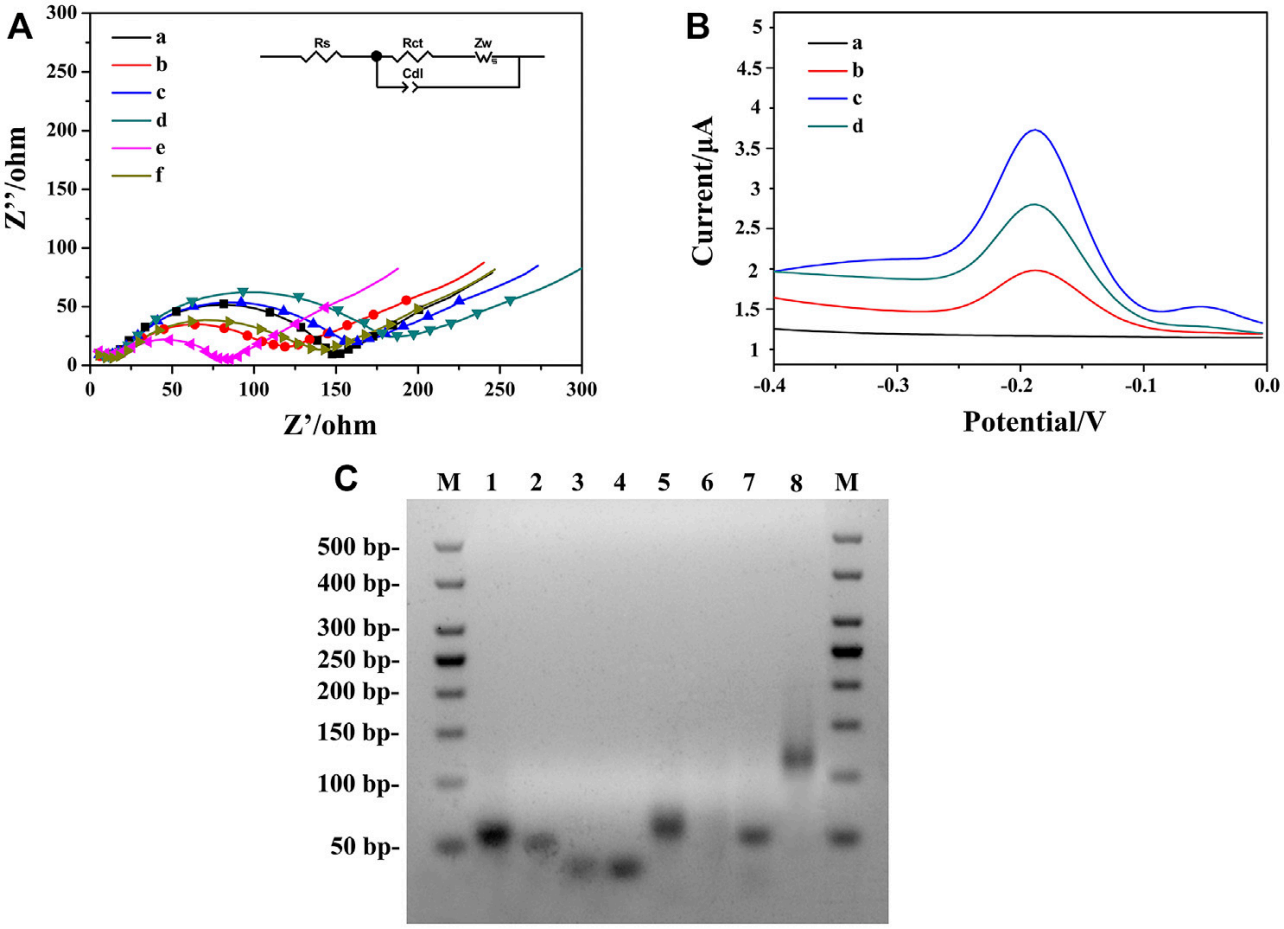
**(A)** Nyquist plots of different modified electrodes were recorded in PBS containing 5.0 mM [Fe(CN)_6_]^3−^/^43−^: (a) bare ITO, (b) AuNPs/ZIF-8/ITO, (c) HP1/AuNPs/ZIF-8/ITO, (d) BSA/HP1/AuNPs/ZIF-8/ITO, (e) ssDNA/HP1/AuNPs/ZIF-8/ITO, (f) HCR/ssDNA/HP1/AuNPs/ZIF-8/ITO. **(B)** DPV signals in PBS at (a) ssDNA/HP1/AuNPs/ZIF-8/ITO, (b) HCR/ssDNA/HP1/AuNPs/ZIF-8/ITO with Thi, (c) solution b added with NADH, Thi, and hemin, (d) HCR/ssDNA/HP1/AuNPs/ZIF-8/ITO formed after the addition of 10^3^ fg ml^−1^ OTA. **(C)** The AGE characterization. Lane M: DNA marker, lanes 1–4: HP, HP1, HP2, and HP3, respectively; lane 5: HP/RNA; lane 6: solution 5 added with DSN; lane 7: ssDNA/HP1; lane 8: ssDNA/HP1/HCR.

To characterize the stepwise modification process of the aptasensor, EIS at each immobilization step was recorded as shown in Figure 4A. Curve a represented the impedance spectrum of ITO, with an electron transfer resistance (R_et_) value around 135.2 Ω. When the AuNPs/ZIF-8 was modified onto the ITO surface, the diameter of semicircle (curve b) decreased since external AuNPs helped enhance the interface electron transfer. After the modification of HP1 through Au–S bond, R_et_ value increased from 96.7 to 133.6 Ω (curve c), which can be explained that the negative-charged HP1 electrostatically repulsed the negative-charged electrochemical probe of [Fe(CN)_6_]^3−^/^4−^, decreasing the electron transfer rate from [Fe(CN)_6_]^3−^/^4−^ to electrode surface. A larger semicircle domain was displayed after the electrode was blocked with 0.25% BSA (curve d). Curve e depicts the hybridization of ssDNA with HP1, by unfolding HP1 from hairpin rigid structure to liner flexible structure, with a very small R_et_ value around 71 Ω. The decrease of R_et_ can be explained in terms of steric effects caused by structural changes of chains. As for electrode treating with HCR, a further increased R_et_ was observed because the exponential negatively charged nucleic acids immobilized onto the sensing interface (Wang X. et al., 2019). According to the characterization of impedance spectra, each step of fabrication was successfully conducted and HCR/ssDNA/HP1/AuNPs/ZIF-8/ITO was proved to be well assembled. The fitted R_et_ values were calculated based on the equivalent circuit, which consisted of four main elements: electrolyte solution resistance (R_s_), R_et_, the double layer capacitance (C_dl_), and Warburg impedance (Z_w_) (inset part in Figure 4A).

Feasibility of the strategy employed for amplifying signals was verified by DPV with different modified electrodes under diverse detection conditions. Figure 4B illustrates the DPV responses of different aptasensors. Curve a corresponded to ssDNA/HP1/AuNPs/ZIF-8/ITO electrode in PBS, in which no visible DPV signals could be seen. It indicated that there was no electrochemical probe Thi on the electrode surface at this time. After HCR structure formed on the electrode surface, Thi could be electrostatically adsorbed on HCR, arousing a low current response (curve b) mainly due to the insulating nature of oligonucleotides. When hemin/G-quadruplex DNAzyme formed with the participation of hemin, an obviously increased peak current was obtained in the presence of 3.0 mM NADH (curve c) due to the synergistic catalytic effect between DNAzyme and NADH. The obtained current was about 2.6 times of that in curve b, illustrating enhanced signal amplification. For comparison, in the presence of 10^3^ fg ml^−1^ target OTA, a weaker DPV signal (curve d) was recorded since the strong affinity between HP and OTA caused less ssDNA to be released.

To further evaluate the feasibility of the aptasensor, AGE analysis was conducted by DNA electrophoresis on various stages of samples (Bai et al., 2021). As shown in Figure 4C, lanes 1 to 4 corresponded to HP, HP1, HP2, and HP3, and four bright bands could be observed respectively. In the absence of OTA, HP hybridized with RNA, which gave a good explanation for a bright band in lane 5, and the height was a little higher than that of HP. After adding DSN in duplex HP/RNA, HP was digested while ssDNA was released. No obvious band was observed in lane 6, which might be explained by the fact that single-stranded ssDNAs with much smaller molecular weights were hard to detect in electrophoresis analysis. Lanes 7 and 8 corresponded to ssDNA/HP1 and ssDNA/HP1/HCR, two bright bands that illustrated the hybridization among ssDNA, HP1, AP, HP2, and HP3 was successful. In addition, lane 7 was found to be a little higher than lane 2, which can be attributed to the combination of ssDNA and HP1. Lane 8 was the highest band in this image, illustrating the large molecule nature of HCR structure. Therefore, the AGE result fully confirmed the strong interactions among these sequences and the feasibility of the sensing strategy in this work.

### Kinetic Study of the Modified Electrode

**FIGURE 5:**
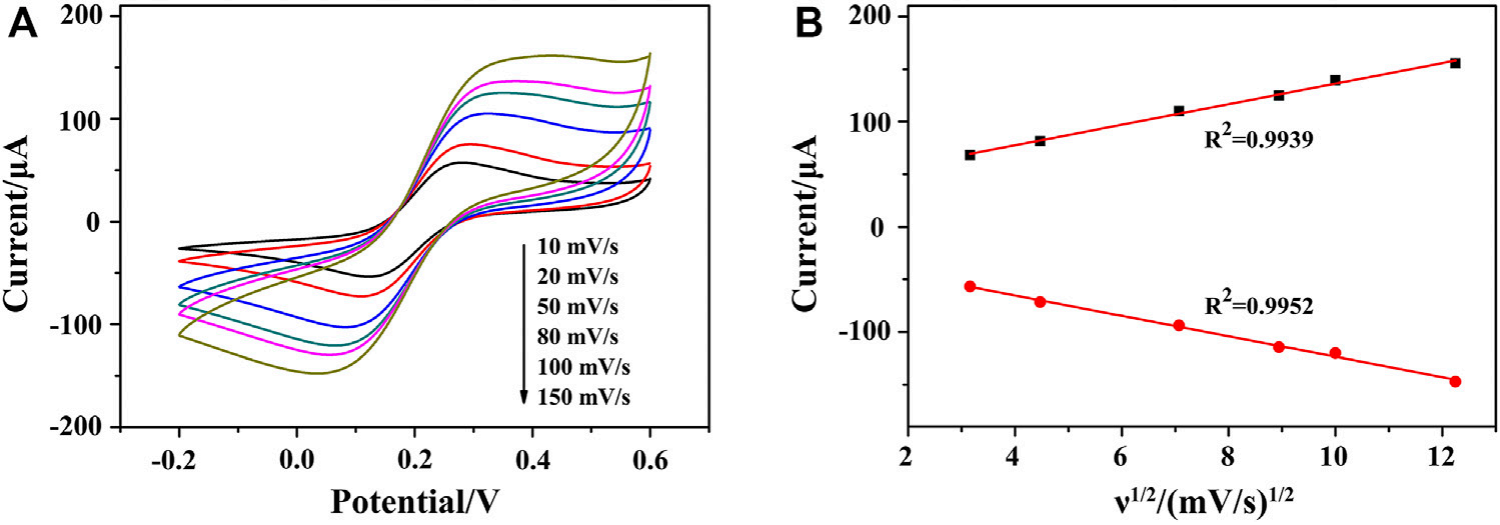
**(A)** CVs of HP1/AuNPs/ZIF-8/ITO at the scan rate of 10, 20, 50, 80, 100, and 150 mV s^−1^ (from internal to external) in PBS solution containing 5.0 mM [Fe(CN)_6_]^3−^/^4−^, respectively. **(B)** The corresponding plot of peak current *vs*. ν^1/2^.

To study the kinetic principle of the electrochemical process, CV curves were recorded at different scan rates (ν) from 10 to 150 mV s^−1^. As shown in Figure 5A, CV curves displayed good symmetry at different scanning rates accompanying with a significant pair of redox peaks, in which [Fe(CN)_6_]^3−^/^4−^ worked as the oxidation–reduction probe. By deducing the relationship between redox peak currents and the square root of scan rate (ν^1/2^), a linear relationship can be acquired, indicating a diffusion-controlled electrochemical process. It meant that a fast electron transfer occurs between the Fe(CN)_6_^4−^ and the electrode surface, demonstrating an excellent conductivity of AuNPs/ZIF-8 and a uniform distribution of AuNPs in pores of ZIF-8 (Figure 5B).

### Optimization of Experimental Parameters

**FIGURE 6:**
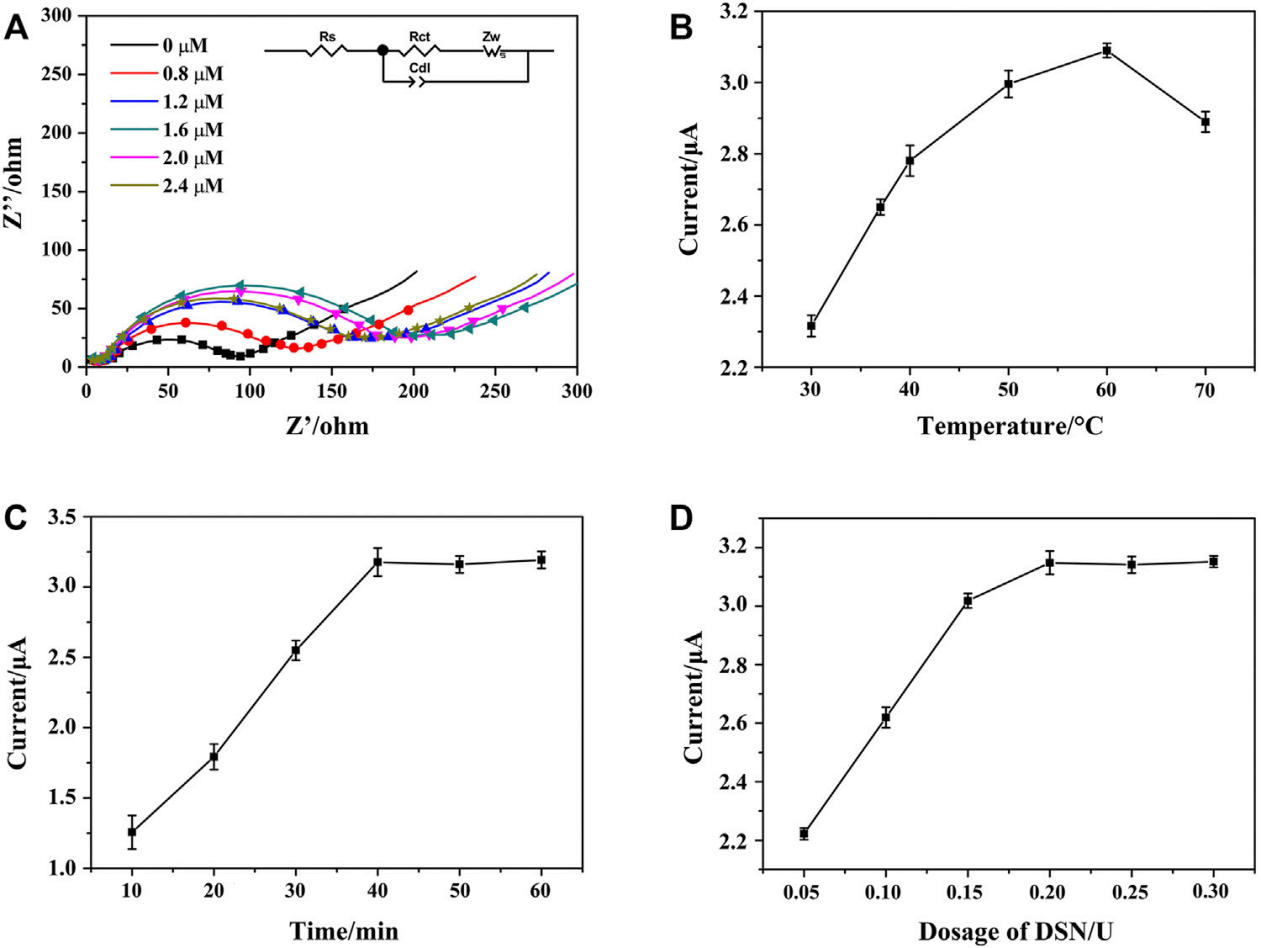
**(A)** Nyquist plots of HP1/AuNPs/ZIF-8/ITO immobilized with different concentrations of HP1, which recorded in PBS containing 5.0 mM [Fe(CN)_6_]^3−^/^4−^. Effects of DSN catalytic nicking reaction parameters of **(B)** reaction temperature, **(C)** reaction time, and **(D)** DSN dosage on the peak current of HCR/ssDNA/HP1/AuNPs/ZIF-8/ITO in the presence of NADH and Thi, respectively.

To improve the performance of the aptasensor, the reaction parameters of HP1 concentration, DSN dosage, DSN cleaving temperature, and reaction time were investigated by EIS and DPV techniques (Li Xiaoyan et al., 2021; Peng et al., 2018; Zhang et al., 2019). As shown in Figure 6A, along with the concentration of HP1 that increased from 0 to 2.4 μM, the detected R_et_ value of HP1/AuNPs/ZIF-8/ITO increased accordingly and the largest value appeared at 1.6 μM. If the immobilized HP1 exceeded 1.6 μM, the R_et_ value seemed decreased a little, which might be ascribed to the excessive HP1 that could not link the limited active sites on AuNPs/ZIF-8/ITO surface. It meant that 1.6 μM of HP1 immobilization was saturated on electrode surface.

DSN could cleave HP in heteroduplex of HP/RNA and thus release the RNA strand for amplification cycle. The reaction temperature, time, and DSN dosage are important for the optimal performance of DSN catalytic reaction, which should be studied and discussed. To maximize the effect of DSN catalysis, reaction time and DSN dosage were fixed at 60 min and 0.3 U, respectively. The peak currents of HCR/ssDNA/HP1/AuNPs/ZIF-8/ITO in the presence of NADH and Thi were recorded. As Figure 6B illustrates, peak current successively increased when the reaction temperature rose from 30 to 60°C, since more and more ssDNA could be released after DSN catalysis. However, the current was observed to decrease at 70°C, which might be due to less stability of DSN and HP/RNA heteroduplex at a higher temperature. In addition, the peak current was found to be increased along with the reaction time that was prolonged from 10 to 40 min, and then reached a plateau. Similarly, the peak current was also found to reach a maximum and steady value at the DSN dosage of 0.2 U. It meant that 40 min of reaction time at 60°C and 0.2 U of DSN dosage were sufficient for the enzyme catalyzed nicking reaction.

### Quantitative Determination of OTA

**FIGURE 7:**
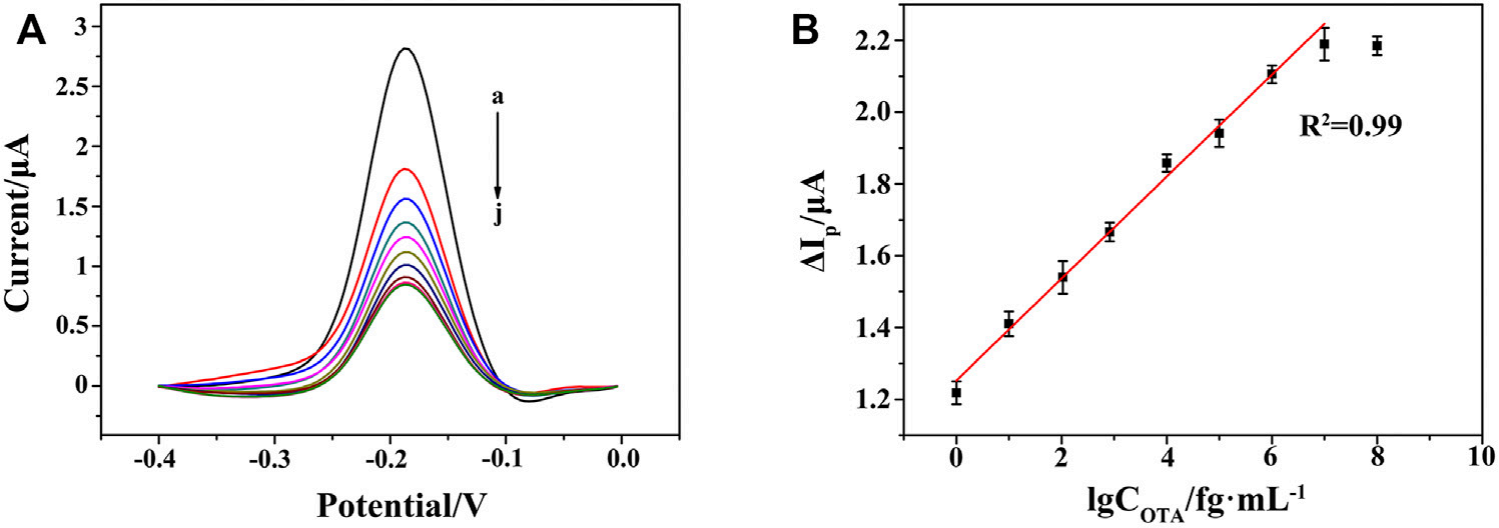
**(A)** DPV responses at the HCR/ssDNA/HP1/AuNPs/ZIF-8/ITO with varying concentrations of OTA from 0 to 10^7^ fg ml^−1^ (curves a to j: 0, 1 fg ml^−1^, 10 fg ml^−1^, 10^2^ fg ml^−1^, 10^3^ fg ml^−1^, 10^4^ fg ml^−1^, 10^5^ fg ml^−1^, 10^6^ fg ml^−1^, 10^7^ fg ml^−1^, 10^8^ fg ml^−1^). **(B)** Linear relationship between the peak current change and the logarithm of OTA concentration from 1 to 10^7^ fg ml^−1^.

Under the optimal conditions, the performance of the aptasensor was evaluated in terms of sensitivity and dynamic response range. In the absence of OTA, the DPV current of Thi was recorded as I_0_. In the presence of OTA, the high infinity of OTA with its aptamer led to the decrease in the amount of HP/RNA, and thus the corresponding current response of Thi (I) became lower. A gradual reduction of DPV current was observed after adding OTA from 1 to 10^7^ fg ml^−1^ (Figure 7A). As shown in Figures 7A,B, good linearity between the peak current change (ΔI_p_ = I_0_−I) and the logarithm of OTA concentration was obtained, with a linear equation of ΔI_p_/μA = 0.14 lgC_OTA_/fg ml^−1^+1.25 (*n* = 5, *R* ^2^ = 0.99). The limit of detection was evaluated to be 0.247 fg ml^−1^ according to 3 σ principle. Compared with the performance of previously reported OTA aptasensors, as listed in Table 2, the proposed aptasensor exhibited a wide linear range by eight orders of magnitude with a low detection limit. The advantages of our work were ascribed to six aspects: 1) the controllable and perfect structured ZIF-8 provided rich active sites and a large surface area for DNA strands modification; 2) the *in situ* implantation of AuNPs significantly enhanced the electron transfer rate; 3) DSN was used for selective digestion of DNA strand in DNA/RNA hybrid, providing a good chance for designing a RNA recycled amplification strategy; 4) the G-quadruplex-hemin assembled HCR nanowire acted as an NADH oxidase to assist the concomitant formation of H_2_O_2_ in the presence of dissolved O_2_; 5) meanwhile, the G-quadruplex-hemin assembled HCR nanowire acted as an HRP-mimicking DNAzyme to catalyze the reduction of the produced H_2_O_2_; 6) with the redox probe Thi as electron mediator, the pseudobienzyme electrocatalytic system combined with the aptamer recognition strategy endowed a dramatically amplified electrochemical aptasensing performance.

**TABLE 2:** Comparisons of analytical performances of different OTA aptasensors

Signal amplification mode	Liner range (fg ml^−1^)	Detection limit (fg ml^−1^)	Ref
RCA	5 × 10^4^–10^8^	104	Hao et al. (2020)
Exo III-assisted cycling	1–10^5^	0.25	Huang et al. (2020)
Exo I + silver metallized aptamer	10^3^–10^8^	700	Suea-Ngam et al. (2019)
G-quadruplex-hemin	10^4^–10^8^	4,280	Shen et al. (2017)
Restriction endonuclease	10^3^–2 × 10^4^	400	Zhang et al. (2012)
HCR-restriction endonuclease-aided DNA walker	10–10^7^	3.3	Wang et al. (2021)
“DSN + G-quadruplex-hemin” dual-enzyme	1–10^7^	0.247	This work

### Selectivity and OTA Detection in Real Samples

**FIGURE 8:**
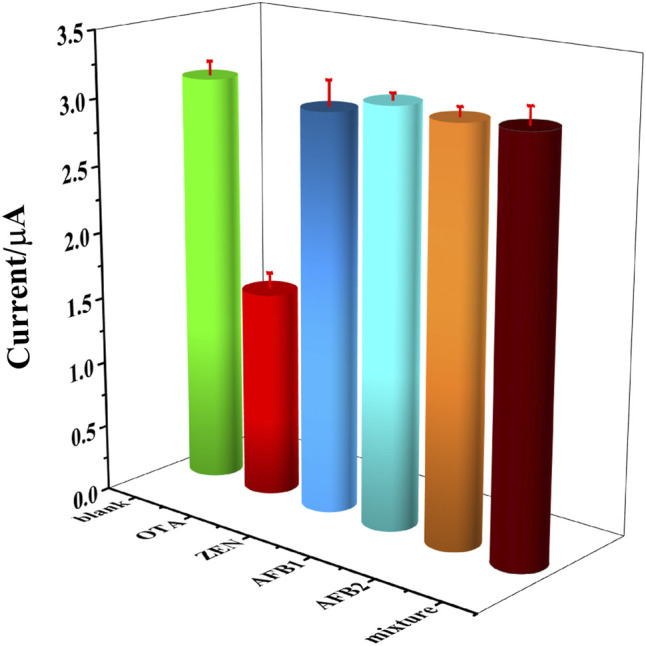
DPV currents recorded in blank solution, 10^3^ fg ml^−1^ of OTA, ZEN, AFB1, AFB2 and the mixture of above interfering substance, respectively.

To evaluate the selectivity of the constructed OTA aptasensing strategy, 10^3^ fg ml^−1^ of zearalenone (ZEN), aflatoxin B1 (AFB1), aflatoxin B2 (AFB2), and the mixture of the aforementioned interfering substances were tested as the normal interferents toward OTA. Herein, blank solution without OTA and 10^3^ fg ml^−1^ of OTA were set as control samples. It could be seen that a significantly decreased peak current was obtained in 10^3^ fg ml^−1^ of OTA solution, while the peak currents detected in interferents were similar to that in blank solution, as could be seen in Figure 8. It could be demonstrated that there is a high selectivity of this OTA aptasensor, which was owing to the high affinity between aptamer and OTA.

The performance of the aptasensor was further evaluated in some OTA real samples. Wheat flour (5 g) purchased from the market was added into 20 ml of acetonitrile–water (v/v = 9:1) mixture, and the supernatant was collected for use after centrifugation and filtration. Wheat extract was diluted with PBS via the volume ratio of 1:9, then 1 ml of OTA standard solutions with the concentration of 10^2^ fg ml^−1^, 10^5^ fg ml^−1^, and 10^8^ fg ml^−1^ were spiked respectively into 9 ml of the aforementioned extractions. As shown in Table 3, the recoveries of the three samples were in the range of 96.8–104.2% with the relative standard deviation (RSD) values of 3.0–5.9%. According to the result, the proposed aptasensing strategy was expected to become a powerful tool for OTA residue detection in real samples with enough precision and accuracy.

**TABLE 3:** The recovery determination in real wheat flour samples

Sample	Added (fg ml^−1^)	Found (fg ml^−1^)	Recovery (%)	RSD (%)
1	102	99.8	99.8	1.6
2	105	9.68 × 104	96.8	5.9
3	108	1.042 × 108	104.2	3.0

## Conclusion

In conclusion, the AuNPs/ZIF-8 with a typical rhombic dodecahedron shape was prepared by *in situ* reduction method with NaBH_4_. A highly sensitive OTA aptasensor was then designed by conducting an enzymatic signal amplification system, accompanying with ZIF-8 materials and the autonomously assembled hemin/G-quadruplex DNAzyme for further signal amplification. BET experiments revealed that the prepared ZIF-8 provided abundant available functional surface for subsequent modification of AuNPs, which is in favor of immobilization of HP1 on AuNPs/ZIF-8/ITO electrode. Owing to the specific ability of DSN to identify and cleave DNA from DNA/RNA heteroduplex, ssDNA thus could be released and to open HP1 assembled on the electrode, which successfully triggered the HCR and further realized the pseudobienzyme electrocatalytic amplification. Catalytic results indicated that this constructed aptasensor exhibited a high sensitivity from 1 to 10^7^ fg ml^−1^ and a low detection limit of 0.247 fg ml^−1^. Furthermore, the aptasensor was proven to be applied in wheat samples, which demonstrated the potential prospects in practical detection.

## Data Availability

The original contributions presented in the study are included in the article/Supplementary Material, further inquiries can be directed to the corresponding author.

## References

[B1] Abrunhosa L. Serra R. Venâncio A. (2002). Biodegradation of Ochratoxin A by Fungi Isolated from Grapes. J. Agric. Food Chem. 50 (25), 7493–7496. 10.1021/jf025747i 12452682

[B2] Armstrong-Price D. E. Deore P. S. Manderville R. A. (2020). Intrinsic "Turn-On" Aptasensor Detection of Ochratoxin A Using Energy-Transfer Fluorescence. J. Agric. Food Chem. 68 (7), 2249–2255. 10.1021/acs.jafc.9b07391 31986034

[B3] Bai Y. Zhang H. Zhao L. Wang Y. Chen X. Zhai H. (2021). A Novel Aptasensor Based on HCR and G-Quadruplex DNAzyme for Fluorescence Detection of Carcinoembryonic Antigen. Talanta 221, 121451. 10.1016/j.talanta.2020.121451 33076074

[B4] Bougrini M. Baraket A. Jamshaid T. Aissari A. E. Bausells J. Zabala M. (2016). Development of A Novel Capacitance Electrochemical Biosensor Based on Silicon Nitride for Ochratoxin A Detection. Sensors Actuators B: Chem. 234, 446–452. 10.1016/j.snb.2016.03.166

[B5] Chen J. Fang Z. Liu J. Zeng L. (2012). A Simple and Rapid Biosensor for Ochratoxin A Based on A Structure-Switching Signaling Aptamer. Food Control 25 (2), 555–560. 10.1016/j.foodcont.2011.11.039

[B6] Cheow L. F. Han J. (2011). Continuous Signal Enhancement for Sensitive Aptamer Affinity Probe Electrophoresis Assay Using Electrokinetic Concentration. Anal. Chem. 83 (18), 7086–7093. 10.1021/ac201307d 21809885 PMC3179849

[B7] Cho Y.-J. Lee D.-H. Kim D.-O. Min W.-K. Bong K.-T. Lee G.-G. (2005). Production of A Monoclonal Antibody against Ochratoxin A and its Application to Immunochromatographic Assay. J. Agric. Food Chem. 53 (22), 8447–8451. 10.1021/jf051681q 16248536

[B8] Cruz-Aguado J. A. Penner G. (2008). Determination of Ochratoxin A with A Dna Aptamer. J. Agric. Food Chem. 56 (22), 10456–10461. 10.1021/jf801957h 18983163

[B9] Duan H. Huang X. Shao Y. Zheng L. Guo L. Xiong Y. (2017). Size-Dependent Immunochromatographic Assay with Quantum Dot Nanobeads for Sensitive and Quantitative Detection of Ochratoxin A in Corn. Anal. Chem. 89 (13), 7062–7068. 10.1021/acs.analchem.7b00869 28573854

[B10] Gao L. Wu Z. Ibrahim A.-R. Zhou S.-F. Zhan G. (2020). Fabrication of Folic Acid-Decorated Hollow ZIF-8/Au/CuS Nanocomposites for Enhanced and Selective Anticancer Therapy. ACS Biomater. Sci. Eng. 6 (11), 6095–6107. 10.1021/acsbiomaterials.0c01152 33449663

[B11] García-Campaña A. M. Lombardo-Agüí M. Arroyo-Manzanares N. Gámiz-Gracia L. Cruces-Blanco C. (2012). XV International Symposium on Luminescence Spectrometry-Biophysical and Analytical Aspects, Extended Abstracts, 19-22 June 2012, Barcelona, Spain-(ISLS 2012). Luminescence 27 (6), 534–572. 10.1002/bio.2432

[B12] Giovannoli C. Passini C. Di Nardo F. Anfossi L. Baggiani C. (2014). Determination of Ochratoxin A in Italian Red Wines by Molecularly Imprinted Solid Phase Extraction and HPLC Analysis. J. Agric. Food Chem. 62 (22), 5220–5225. 10.1021/jf5010995 24823614

[B13] Han B. Zhao C. Yin J. Wang H. (2012). High Performance Aptamer Affinity Chromatography for Single-step Selective Extraction and Screening of Basic Protein Lysozyme. J. Chromatogr. B 903, 112–117. 10.1016/j.jchromb.2012.07.003 22841745

[B14] Hao L. Wang W. Shen X. Wang S. Li Q. An F. (2020). A Fluorescent DNA Hydrogel Aptasensor Based on the Self-Assembly of Rolling Circle Amplification Products for Sensitive Detection of Ochratoxin A. J. Agric. Food Chem. 68 (1), 369–375. 10.1021/acs.jafc.9b06021 31829586

[B15] Hao Y.-B. Shao Z.-S. Cheng C. Xie X.-Y. Zhang J. Song W.-J. (2019). Regulating Fluorescent Aptamer-Sensing Behavior of Zeolitic Imidazolate Framework (ZIF-8) Platform via Lanthanide Ion Doping. ACS Appl. Mater. Inter. 11 (35), 31755–31762. 10.1021/acsami.9b12253 31393692

[B16] Huang X.-B. Wu S.-H. Hu H.-C. Sun J.-J. (2020). AuNanostar@4-MBA@Au Core-Shell Nanostructure Coupled with Exonuclease III-Assisted Cycling Amplification for Ultrasensitive SERS Detection of Ochratoxin A. ACS Sens. 5 (8), 2636–2643. 10.1021/acssensors.0c01162 32786384

[B17] Keskin S. (2011). Atomistic Simulations for Adsorption, Diffusion, and Separation of Gas Mixtures in Zeolite Imidazolate Frameworks. J. Phys. Chem. C 115 (3), 800–807. 10.1021/jp109743e

[B18] Lee H. J. Ryu D. (2015). Significance of Ochratoxin A in Breakfast Cereals from the United States. J. Agric. Food Chem. 63 (43), 9404–9409. 10.1021/jf505674v 25661245

[B19] Lee H. J. Ryu D. (2017). Worldwide Occurrence of Mycotoxins in Cereals and Cereal-Derived Food Products: Public Health Perspectives of Their Co-occurrence. J. Agric. Food Chem. 65 (33), 7034–7051. 10.1021/acs.jafc.6b04847 27976878

[B20] Li X. Ni X. Cui F. Qiu Q. Chen X. Huang H. (2021). A Logic Dual-Channel Detection of Hox Transcript Antisense Intergenic RNA Using Graphene Switch and Padlock Probe-Based Exponential Rolling Circle Amplification Assay. Sensors Actuators B: Chem. 340, 129931. 10.1016/j.snb.2021.129931

[B21] Li Y. Li S. Bao M. Zhang L. Carraro C. Maboudian R. (2021). Pd Nanoclusters Confined in ZIF-8 Matrixes for Fluorescent Detection of Glucose and Cholesterol. ACS Appl. Nano Mater. 4 (9), 9132–9142. 10.1021/acsanm.1c01712

[B22] Manna B. Raj C. R. (2018). Nanostructured Sulfur-Doped Porous Reduced Graphene Oxide for the Ultrasensitive Electrochemical Detection and Efficient Removal of Hg(II). ACS Sust. Chem. Eng. 6 (5), 6175–6182. 10.1021/acssuschemeng.7b04884

[B23] Park K. S. Ni Z. Cote A. P. Choi J. Y. Huang R. Uribe-Romo F. J. (2006). Exceptional Chemical and Thermal Stability of Zeolitic Imidazolate Frameworks. Proc. Natl. Acad. Sci. 103 (27), 10186–10191. 10.1073/pnas.0602439103 16798880 PMC1502432

[B24] Peng G. Li X. Cui F. Qiu Q. Chen X. Huang H. (2018). Aflatoxin B1 Electrochemical Aptasensor Based on Tetrahedral DNA Nanostructures Functionalized Three Dimensionally Ordered Macroporous MoS2-AuNPs Film. ACS Appl. Mater. Inter. 10 (21), 17551–17559. 10.1021/acsami.8b01693 29733573

[B25] Pittet A. Royer D. (2002). Rapid, Low Cost Thin-Layer Chromatographic Screening Method for the Detection of Ochratoxin A in Green Coffee at a Control Level of 10 μg/kg. J. Agric. Food Chem. 50 (2), 243–247. 10.1021/jf010867w 11782189

[B26] Qiu X. Zhang H. Yu H. Jiang T. Luo Y. (2015). Duplex-Specific Nuclease-Mediated Bioanalysis. Trends Biotechnol. 33 (3), 180–188. 10.1016/j.tibtech.2014.12.008 25640199

[B27] Salandari-Jolge N. Ensafi A. A. Rezaei B. (2021). Ultra-Sensitive Electrochemical Aptasensor Based on Zeolitic Imidazolate Framework-8 Derived Ag/Au Core-Shell Nanoparticles for Mercury Detection in Water Samples. Sensors Actuators B: Chem. 331, 129426. 10.1016/j.snb.2020.129426

[B28] Shen P. Li W. Liu Y. Ding Z. Deng Y. Zhu X. (2017). High-Throughput Low-Background G-Quadruplex Aptamer Chemiluminescence Assay for Ochratoxin A Using A Single Photonic Crystal Microsphere. Anal. Chem. 89 (21), 11862–11868. 10.1021/acs.analchem.7b03592 28988477

[B29] Suea-Ngam A. Howes P. D. Stanley C. E. DeMello A. J. (2019). An Exonuclease I-Assisted Silver-Metallized Electrochemical Aptasensor for Ochratoxin A Detection. ACS Sens. 4 (6), 1560–1568. 10.1021/acssensors.9b00237 31062585

[B30] Sun X. Chen H. Wang S. Zhang Y. Tian Y. Zhou N. (2018). Electrochemical Detection of Sequence-specific DNA Based on Formation of G-Quadruplex-Hemin through Continuous Hybridization Chain Reaction. Analytica Chim. Acta 1021, 121–128. 10.1016/j.aca.2018.02.076 29681278

[B31] Sun Y. Shi J. Zhang S. Wu Y. Mei S. Qian W. (2019). Hierarchically Porous and Water-Tolerant Metal-Organic Frameworks for Enzyme Encapsulation. Ind. Eng. Chem. Res. 58 (28), 12835–12844. 10.1021/acs.iecr.9b02164

[B32] Sun Z. Lv J. Liu X. Tang Z. Wang X. Xu Y. (2018). Development of A Nanobody-AviTag Fusion Protein and its Application in A Streptavidin-Biotin-Amplified Enzyme-Linked Immunosorbent Assay for Ochratoxin A in Cereal. Anal. Chem. 90 (17), 10628–10634. 10.1021/acs.analchem.8b03085 30092629 PMC7307665

[B33] Torad N. L. Hu M. Kamachi Y. Takai K. Imura M. Naito M. (2013). Facile Synthesis of Nanoporous Carbons with Controlled Particle Sizes by Direct Carbonization of Monodispersed ZIF-8 Crystals. Chem. Commun. 49 (25), 2521. 10.1039/c3cc38955c 23423451

[B34] Tuncel D. Ökte A. N. (2021). Improved Adsorption Capacity and Photoactivity of ZnO-ZIF-8 Nanocomposites. Catal. Today 361, 191–197. 10.1016/j.cattod.2020.04.014

[B35] Wang P.-L. Xie L.-H. Joseph E. A. Li J.-R. Su X.-O. Zhou H.-C. (2019a). Metal-Organic Frameworks for Food Safety. Chem. Rev. 119 (18), 10638–10690. 10.1021/acs.chemrev.9b00257 31361477

[B36] Wang X. Shan Y. Gong M. Jin X. Lv L. Jiang M. (2019b). A Novel Electrochemical Sensor for Ochratoxin A Based on the Hairpin Aptamer and Double Report DNA via Multiple Signal Amplification Strategy. Sensors Actuators B: Chem. 281, 595–601. 10.1016/j.snb.2018.10.148

[B37] Wang Y. Li X. Waterhouse G. I. N. Zhou Y. Yin H. Ai S. (2019c). Photoelectrochemical Biosensor for Protein Kinase A Detection Based on Carbon Microspheres, Peptide Functionalized Au-ZIF-8 and TiO2/g-C3n4. Talanta 196, 197–203. 10.1016/j.talanta.2018.12.035 30683351

[B38] Wang Y. Song W. Zhao H. Ma X. Yang S. Qiao X. (2021). DNA Walker-Assisted Aptasensor for Highly Sensitive Determination of Ochratoxin A. Biosens. Bioelectron. 182, 113171. 10.1016/j.bios.2021.113171 33773380

[B39] Yang J. Zhang F. Lu H. Hong X. Jiang H. Wu Y. (2015). Hollow Zn/Co ZIF Particles Derived from Core-Shell ZIF-67@ZIF-8 as Selective Catalyst for the Semi-hydrogenation of Acetylene. Angew. Chem. Int. Ed. 54 (37), 10889–10893. 10.1002/anie.201504242 26333054

[B40] Yang Y.-J. Zhou Y. Xing Y. Zhang G.-M. Zhang Y. Zhang C.-H. (2019). A Label-free Aptasensor Based on Aptamer/NH2 Janus Particles for Ultrasensitive Electrochemical Detection of Ochratoxin A. Talanta 199, 310–316. 10.1016/j.talanta.2019.02.015 30952263

[B41] Ying Z.-M. Xiao H.-Y. Tang H. Yu R.-Q. Jiang J.-H. (2018). Light-up RNA Aptamer Enabled Label-free Protein Detection via A Proximity Induced Transcription Assay. Chem. Commun. 54 (64), 8877–8880. 10.1039/C8CC04498H 30043035

[B42] Yuan Y. Gao M. Liu G. Chai Y. Wei S. Yuan R. (2014). Sensitive Pseudobienzyme Electrocatalytic DNA Biosensor for Mercury(II) Ion by Using the Autonomously Assembled Hemin/G-Quadruplex DNAzyme Nanowires for Signal Amplification. Analytica Chim. Acta 811, 23–28. 10.1016/j.aca.2013.11.051 24456590

[B43] Zhang H. Fan M. Jiang J. Shen Q. Cai C. Shen J. (2019). Sensitive Electrochemical Biosensor for MicroRNAs Based on Duplex-specific Nuclease-Assisted Target Recycling Followed with Gold Nanoparticles and Enzymatic Signal Amplification. Analytica Chim. Acta 1064, 33–39. 10.1016/j.aca.2019.02.060 30982515

[B44] Zhang J. Chen J. Zhang X. Zeng Z. Chen M. Wang S. (2012). An Electrochemical Biosensor Based on Hairpin-DNA Aptamer Probe and Restriction Endonuclease for Ochratoxin A Detection. Electrochemistry Commun. 25, 5–7. 10.1016/j.elecom.2012.09.006

[B45] Zhang K. Kirlikovali K. O. Varma R. S. Jin Z. Jang H. W. Farha O. K. (2020). Covalent Organic Frameworks: Emerging Organic Solid Materials for Energy and Electrochemical Applications. ACS Appl. Mater. Inter. 12 (25), 27821–27852. 10.1021/acsami.0c06267 32469503

[B46] Zhang K. Schaab M. R. Southwood G. Tor E. R. Aston L. S. Song W. (2017). A Collaborative Study: Determination of Mycotoxins in Corn, Peanut Butter, and Wheat Flour Using Stable Isotope Dilution Assay (SIDA) and Liquid Chromatography-Tandem Mass Spectrometry (LC-MS/MS). J. Agric. Food Chem. 65 (33), 7138–7152. 10.1021/acs.jafc.6b04872 27983809

[B47] Zhang Y. Li Q. Liu C. Shan X. Chen X. Dai W. (2018). The Promoted Effect of a Metal-Organic Frameworks (ZIF-8) on Au/TiO2 for CO Oxidation at Room Temperature Both in Dark and under Visible Light Irradiation. Appl. Catal. B: Environ. 224, 283–294. 10.1016/j.apcatb.2017.10.027

[B48] Zhang Z. Xian S. Xi H. Wang H. Li Z. (2011). Improvement of CO2 Adsorption on ZIF-8 Crystals Modified by Enhancing Basicity of Surface. Chem. Eng. Sci. 66 (20), 4878–4888. 10.1016/j.ces.2011.06.051

[B49] Zhang Z. Zhang J. Liu J. Xiong Z. Chen X. (2016). Selective and Competitive Adsorption of Azo Dyes on the Metal-Organic Framework ZIF-67. Water Air Soil Poll 227 (12). 10.1007/s11270-016-3166-7

